# Synergistic effects of tung oil and heat treatment on physicochemical properties of bamboo materials

**DOI:** 10.1038/s41598-019-49240-8

**Published:** 2019-09-06

**Authors:** Tong Tang, Bo Zhang, Xianmiao Liu, Wenbo Wang, Xiufang Chen, Benhua Fei

**Affiliations:** 1 Key Laboratory of Bamboo and Rattan Science and Technology of the State Forestry Administration, Department of Bio-materials, International Centre for Bamboo and Rattan, Futong Dong Dajie, Chaoyang District, Beijing 100102 China; 2 Qingdao Institute of Bioenergy and Bioprocess Technology, Chinese Academy of Sciences, Songling road, Qingdao, 266101 Shandong China; 3 Research Institute of Forestry, Chinese Academy of Forestry, Xiangshan Road, Haidian District, Beijing 100091 China

**Keywords:** Biophysical chemistry, Chemical modification, Composites

## Abstract

The search for green and sustainable modification method to produce durable bamboo materials remains a challenge in industry. Here, heat treatment in tung oil at 100–200 °C was employed to modify bamboo materials. Oil permeation and distribution in the structure of bamboo samples during heat treatment were explored. The synergistic effects of tung oil and heat treatment on the chemical, physical and mechanical properties of bamboo materials, and their mutual relationships were also investigated in detail. Results showed that the tung oil heat treated bamboo not only had an enhanced hydrophobic property and dimensional stability, improved fungi resistance, but also displayed good mechanical performance. Compared with the untreated sample, the water-saturated swelling reduced from 3.17% to 2.42% for the sample after oil heat treatment at 200 °C, and the contact angles of the sample after oil heat treatment at 200 °C can keep >100° after 300 s in radial direction. Such improvement can be attributed to changes of chemical components, increased crystallinity structure, and the formation of oily films inside or over the bamboo surface. Therefore, tung oil heat treatment can be a highly promising technology for bamboo modification in the industry.

## Introduction

The severe depletion of forests has ignited efforts to utilize non-wood materials to meet the huge demand of engineering material in our industrial society. As is known, bamboo is one of the most abundant biomass resources. In total, there are an estimated 31.5 million hectares of bamboo forest in the world^1^. Due to the desirable features, such as environmental adaptability, short growth cycle, high yield, light weight and excellent mechanical properties, bamboo is an essential alternative to wood as a renewable raw material. It can be widely used in construction, building facade, decoration, furniture, and household wares^2–6^. However, bamboo has some inherent drawbacks like hydrophilic nature, dimensional instability and low resistance to decay, which would greatly shorten its service life^7–9^. Bamboo mainly consists of parenchyma cells and vascular bundles, made up of longitudinally oriented cellulose fibers embedded in an amorphous matrix of hemicellulose and lignin^10,11^. The abundant presence of hydroxyl groups and hierarchical pore structure would make it easy for bamboo to absorb water from the surrounding environment. The moisture variation in the cell walls would bring about swelling and shrinking, and ultimately cause serious deformation or cracks of bamboo. Moreover, owing to this affinity to water, bamboo is prone to mildew damage, resulting in its natural degradation. Consequently, it is essential to develop durable bamboo materials to more effectively utilize bamboo.

Some efforts have been devoted to modifying bamboo to improve the less-beneficial features, including hydrophilic nature, dimensional instability and low fungi resistance^9,12^. Traditional techniques largely focused on the use of harmful preservatives, creating unexpected environmental hazards. From the standpoint of cost-effective, eco-friendly and sustainable chemistry, heat treatment is considered to be the most effective approach to tackle this issue. It is well-accepted that heat treatment of wood can not only modify its weather resistance, hygroscopic properties and durability, but also reduce swelling and surface roughness, and thus has been widely used in the wood industry^13^. However, as wood was usually treated at high temperature for a long time to obtain better performance, there remained great concern about the compromise of its mechanical strength in some cases^14–16^. Recently, heat treatment in oil medium was reported to be a green and excellent method to improve wood properties. Since then, much attention has been given to studying the effects of oil heat treatment on the chemical and mechanical properties of wood and improving wood properties by heat treatment with various industrial vegetable oils, such as linseed, palm, coconut, rapeseed and soya^17–21^. However, there are limited studies on bamboo modification by heat treatment in oil medium. Although bamboo and wood have similar chemical composition, the structure of bamboo is quite different from that of wood, which is a comparatively heterogeneous structure with a pronounced radial density gradient. Therefore, the methods of wood modification can not be directly applied to bamboo.

Tung oil, known as the China wood oil, has been extensively used in China to protect wood furniture and construction from fungi decay for over thousands years^22^. Tung oil mainly contains unsaturated fatty acids of α-eleostearic acid (77–82%), oleic acid (3.5–12.7%) and linoleic acid (8–10%)^23^. Due to the presence of these components, tung oil is endowed with excellent resistance to water, mold, bacteria, yellowing and darkening^24^. These highly unsaturated conjugated systems of tung oil could lead to the polymerization and good dynamic mechanical properties as well as thermal stability at room temperature. When the unsaturated oil is sprayed or coated on the surface of wood, it will be oxidized by oxygen and polymerized to form a protective oily film over the wood surface, so as to effectively prevent water uptake^25^. Similarly, heat treatment in tung oil would be effective to modify bamboo materials, which could improve the bamboo properties by synergetic the effect of tung oil and heat treatment. Specifically, heat treatment could reduce the xylan in hemicellulose and greatly lower the content of hydrophilic groups, while tung oil isolate oxygen from the treated specimen, slowing down the degradation of certain biomass constituents^20^. For example, Yang *et al*. compared different thermal modification media (air, nitrogen and linseed oil) in terms of influence on the dimensional stability of moso bamboo, and found that linseed oil heat treated bamboo had a better dimensional performance^26^. However, there is a general lack in the literature of more comprehensive studies on the chemical, surface wettability, fungi resistance and mechanical properties of tung oil heat treated bamboo materials, and their mutual relationships. Furthermore, in rare of the reported studies were explored about tung oil permeation and distribution in the structure of bamboo samples during heat treatment.

Our objective, therefore, is to explore the synergistic effects of tung oil and heat treatment on the chemical, surface wettability, fungi resistance and mechanical properties of moso bamboo, and their mutual relationships. The influence of tung oil heat treatment at different temperatures (23–200 °C) on the chemical composition, morphology, cellulose crystalline structure and various properties of bamboo materials were studied in detail. Oil permeation behavior was also investigated by the staining method. Oil distribution in the structure of bamboo was analyzed by confocal laser scanning microscopy (CLSM). This study provides relatively comprehensive information on the tung oil heat treated bamboo for industrial applications.

## Results and Discussion

### Fabricating process

**Figure 1:**
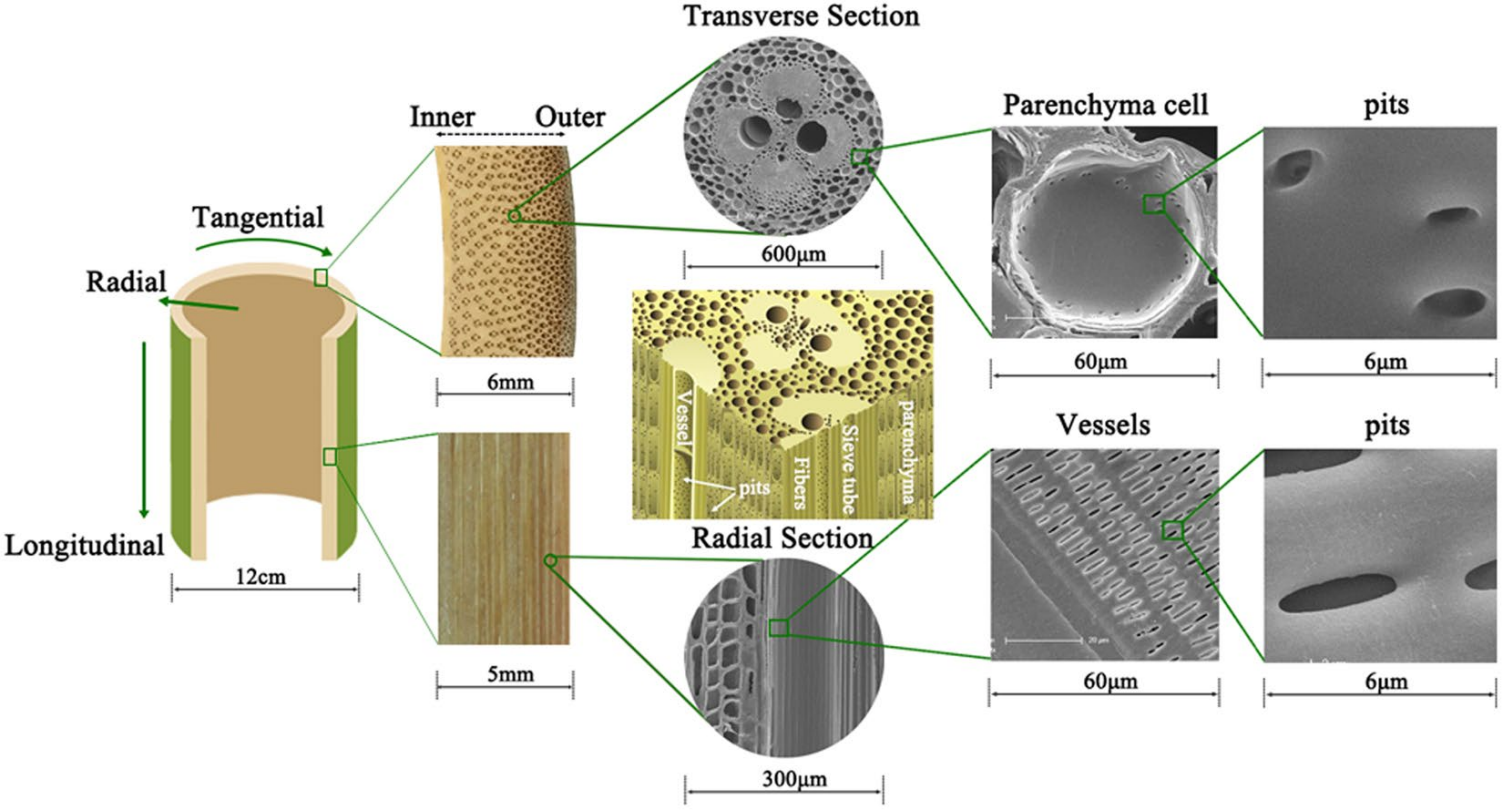
Schematics of the heterogeneous structure of bamboo.

It is known that bamboo has a comparatively heterogeneous structure as a result of natural evolution over time, creating a characteristic radial density gradient of structure, as shown in Fig. 1. Moso bamboo, tissue mainly consists of parenchyma cells, vascular bundles composed of metaxylem vessels, sieve tubes and fibers. On an average, a culm generally consists of 52% parenchyma cells, 40% fibers and 8% conducting tissue (vessels and sieve tubes)^27,28^. The well-developed porous structure would greatly facilitate the diffusion of oil into the cell wall when bamboo samples were immersed in tung oil.

**Figure 2:**
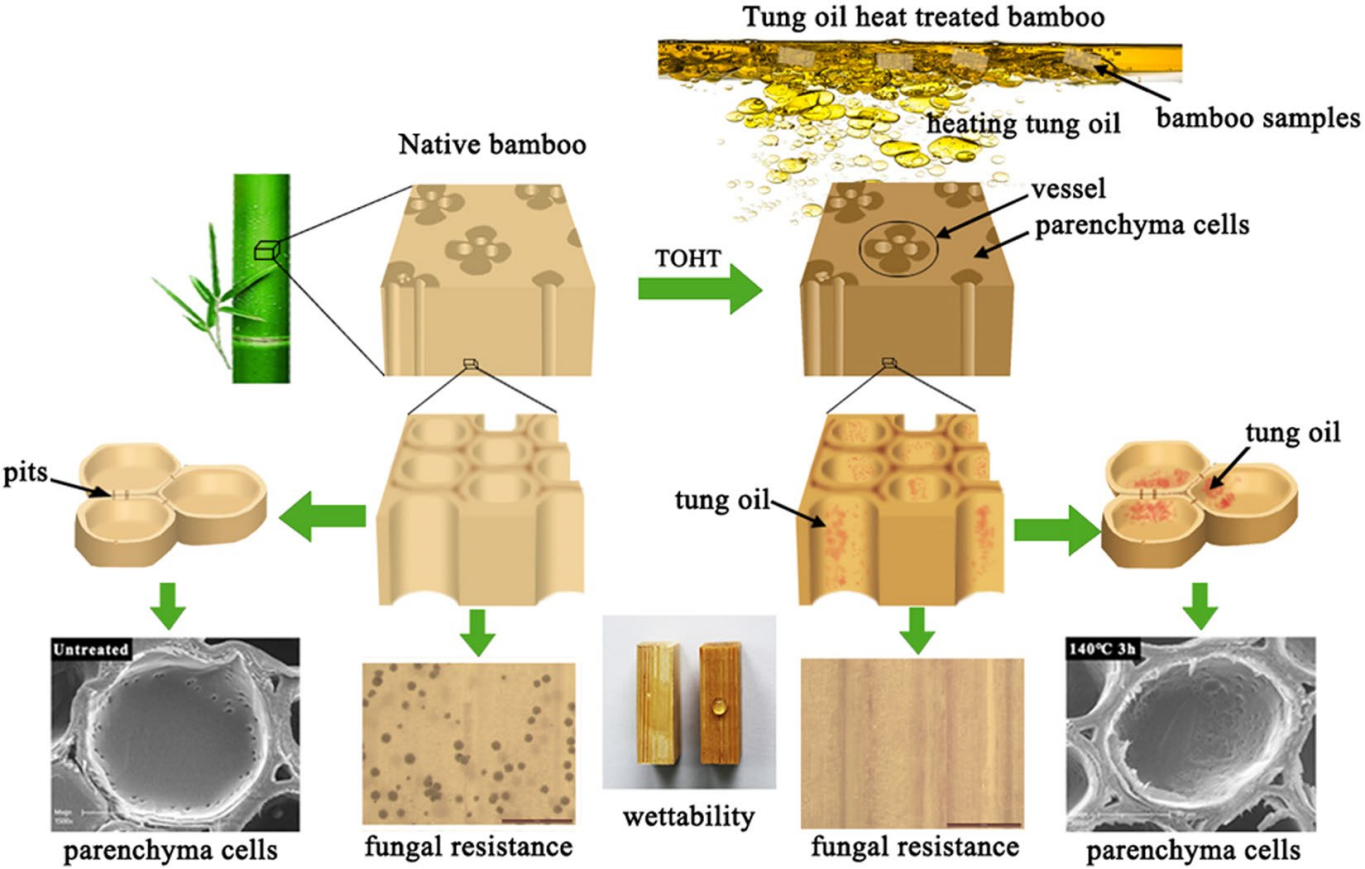
Schematic illustration of the bamboo samples after heat treatment in tung oil.

The process used to fabricate heat treated bamboo in tung oil is schematically illustrated in Fig. 2. Moso bamboo is a typical hierarchical porous material with interconnected three-dimensional network structure. Conducting tissue including vessels and sieve tubes has the largest conduit pores in the bamboo with a size of ~100 μm in diameter and 0.5–0.9 mm in length. No obvious partition appears in the longitudinal direction of the conducting tissue, which is separated by the perforation plates. Parenchyma cells have a relatively closed lumen with a medium size of 14–40 μm in diameter and 0.8–1.6 μm in length, whereas fibers have the smallest pores with a size of ~100 nm in diameter and 1.6–3.1 mm in length. The parenchyma cells and vascular bundles are connected with many pits, which are distributed in their internal walls with a size ranging from sub-microns to a few microns. The abundant pits in the bamboo scaffold would facilitate sufficient interior diffusion of oil into the cell wall during heat treatment in tung oil. Conducting tissue with high interconnectivity provides the main pathway for oil penetration in the longitudinal direction. By contrast, little tissue with straight conduits and high interconnectivity can be found in the radial and tangential directions. Thus, the penetration of oil into the interior of bamboo from the surface in the radial and tangential directions is primarily accomplished through pits^29,30^.

As such, once the bamboo samples were impregnated with tung oil at a certain temperature, most of tung oil firstly permeated through the conducting tissue in the longitudinal direction, and then gradually diffused into adjacent tissues through pits. In addition, a small portion of tung oil also entered through the parenchyma cells and fibers from the bamboo surface. Due to size and connectivity difference, tung oil entered through the conducting tissue had the highest permeability, followed by the parenchyma cells and then the fibers. With the extension of treating time, tung oil gradually achieved fully infiltration into the bamboo samples through pits in the tissue. Upon completion of heat treatment, the bamboo samples were removed from the tung oil solution and cooled to room temperature. Subsequently, tung oil polymerized to form a protective oily film over the pore wall inside the bamboo structure and the surface of the samples.

During the heating process, the bamboo samples became to fill with tung oil, which served as a heating medium to transfer heat to bamboo readily and equally. Owing to the low boiling point, some of the water molecules in the bamboo were evaporated and expelled from the samples, gradually replaced by tung oil. Simultaneously, heat treatment at a high temperature can result in partial degradation of amorphous carbohydrates such as hemicellulose and cause rearrangement of the crystalline region of cellulose, thus forming a more stable structure^31,32^. Such improvement in chemical structure combined with the formation of protective oily films would endow bamboo with some excellent properties for wide application in the floorings and outdoor.

### Oil distribution

**Figure 3:**
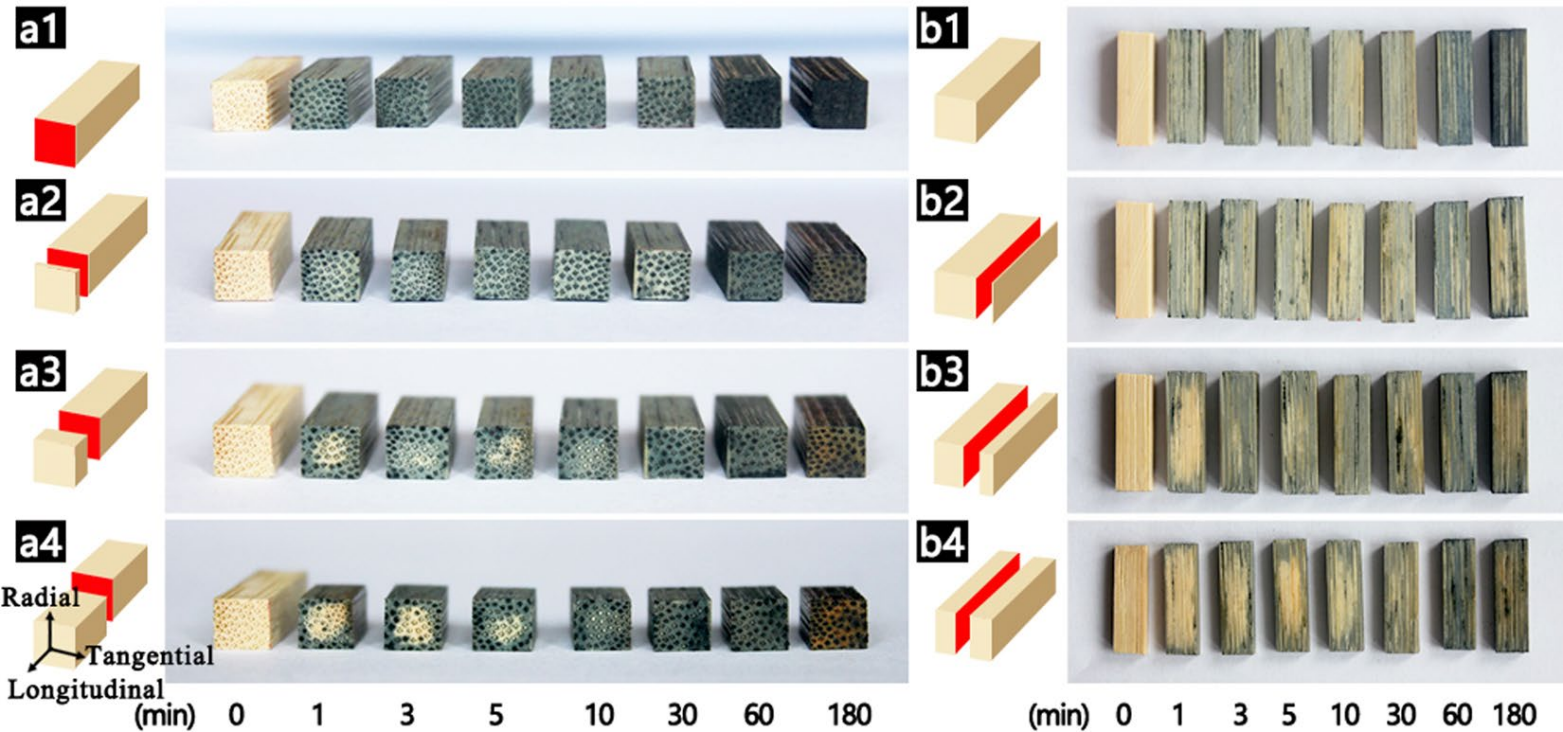
(**a**) Images of the bamboo samples after heat treatment in tung oil and Sudan black solution in transverse section, (a1) surface, (a2) removed 1 mm from surface, (a3) removed 5 mm from surface, (a4) removed 10 mm from surface. (**b**) Images of bamboo in radial section, (b1) surface, (b2) removed 0.5 mm from surface, (b3) removed 1.25 mm from surface, (b4) removed 2.5 mm from surface.

To investigate the tung oil permeation of bamboo samples during heat treatment, control experiments using stain to mark tung oil were carried out. The bamboo samples were thermally treated in tung oil solution mixed with Sudan black dye at 140 °C for different periods of time. Figure 3 displayed the photographs of oil heat treated bamboo samples dyed by Sudan black at different times. The bamboo samples became darker with the increase of treatment time, indicating that tung oil was gradually diffused into the interior of bamboo. At the treating time of 1 min, the sample surface became dark, and the vascular bundles were the darkest (see Fig. 3a1). From the transverse section of different locations (see Fig. 3a2–a4), it was observed that the color of the transverse section of 1 mm location was lighter than the surface, whereas the color change of vascular bundles in the transverse section was not obvious, indicating that tung oil penetrated with the fastest rate into the interior of bamboo through vessels and sieve tubes. In addition, the dark area of the transverse sections gradually reduced from the surface to center, but some vascular bundles in the transverse section of center location still showed dark (Fig. 3a4), supporting the speculation that tung oil infiltration through vessels and sieve tubes was the fastest. With the treatment process continued, the dark area of bamboo samples in the transverse section of center location gradually enlarged, and stabilized after treated for 30–60 min, which suggested that tung oil not only infiltrated from outside to inside, but also easily diffused interior. After the oil entered the vascular bundles and parenchyma cells from the surface, it was further diffused to the adjacent tissues through pits, and finally filled the cell interstices. Since pits are small, the interior diffusion was relatively slow. A comparison between the transverse section of different locations after the first 30 min of treatment also revealed that the permeability of tung oil in the longitudinal direction was remarkably faster than that in the radial direction. The reason may be related to the presence of conducting tissue combined with pits in the longitudinal direction, but only small pits in the radial direction. The results were further supported by the pattern of color change in the radial section, as shown in Fig. 3b1–b4. Thus, it can be inferred that during the process of oil heat treatment of bamboo, most of tung oil permeated the samples along the longitudinal direction through extensive vessels and sieve tubes, and then diffused into the interior by pits. A small part of tung oil permeated the samples along the radial direction through pits.

### Morphology and structure

The weight percent gains (WPG) of bamboo samples after tung oil heat treatment was investigated and the results were shown in Table S1. There was a gradual increase of weight loss as the control samples treated in the air were exposed to rising temperature, probably due to dehydration and degradation of amorphous carbohydrates at high temperature. For the bamboo sample after oil heat treatment at 23 °C, it displayed a WPG of 3.81 wt%, which was directly related to the tung oil content. When the bamboo was heated in tung oil at 100 °C, the modified sample exhibited a WPG of 12.64 wt%, compared to the control sample after heat treatment in the air at the same temperature. The WPG variation proved that tung oil was successfully incorporated into the bamboo. With further heating temperature rising up to 200 °C, the WPG of tung oil gradually reduced, possibly due to the shrinkage of pits after oil treatment at high temperature, which lead to the decline of oil retention capacity of treated samples. The elemental analysis was employed to investigate the change of elemental compositions after oil heat treatment. The modified samples mainly consisted of carbon, oxygen and hydrogen elements. The O/C ratio of the bamboo samples after oil heat treatment decreased as the heating temperature went up (Table S2). A gradual decrease in the tung oil content was also noted with the increase of heating temperature in the range of 100–200 °C. However, tung oil has a much lower ratio O/C than untreated bamboo. It mostly contains α-eleostearic acid (C_18_H_30_O_2_), oleic acid (C_18_H_34_O_2_) and linoleic acid (C_18_H_32_O_2_). Thus, the reduction of O/C ratio in the oil heat treated sample was mainly due to the removal of water or oxygen-containing groups such as hydroxyl groups by heat treatment, supporting the preferential degradation of amorphous carbohydrates such as hemicellulose at high temperature^33^.

**Figure 4:**
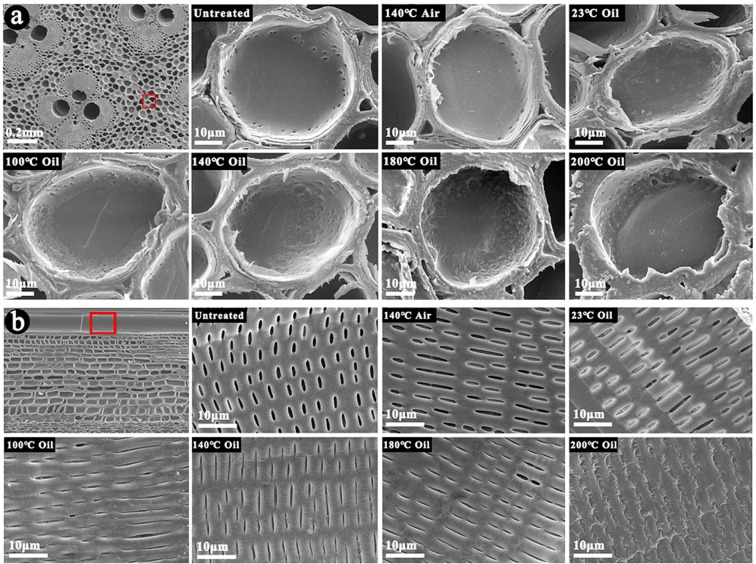
SEM images of different bamboo samples. (**a**) Parenchyma cells in transverse section, (**b**) pits at the radial wall of metaxylem vessels.

**Figure 5:**
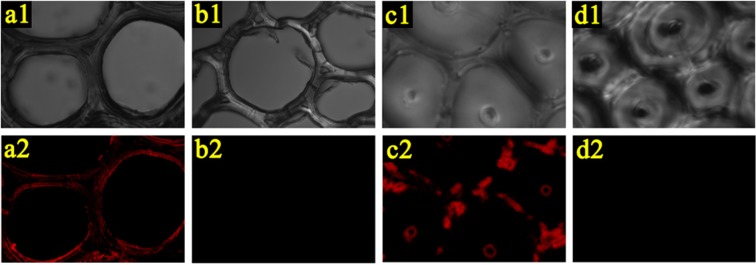
CLSM images of bamboo in transverse section. (**a**,**b**) Parenchyma cells, (**a**) the bamboo sample after oil heat treatment, (**b**) untreated bamboo. (**c**,**d**) Fibers, (**c**) the bamboo sample after oil heat treatment, (**d**) untreated bamboo.

The distribution of tung oil in the bamboo on the microscopic level and the change of bamboo morphology after oil heat treatment were analyzed by scanning electron microscopy (SEM). Figure 4 displayed the representative SEM images of the bamboo samples after oil heat treatment, with the untreated sample and the sample after heat treatment in air as references. The SEM images of the bamboo samples after oil heat treatment confirmed that thin oily layers were formed in the wall surface of parenchyma cells and vessels, and the bamboo structure was mainly retained during treatment. The surface morphology of the parenchyma cells in the transverse section was given in Fig. 4a. It was observed that the bamboo sample after oil treatment at 23 °C had oily films on the surface of parenchyma cells, and the pits in the parenchyma cell were partly or entirely covered with tung oil. For comparison, the lumens of parenchyma cells in the untreated sample and the sample after heat treatment in air were relatively clean and smooth with clearly visible pits. Similar phenomenon was also found in the vessels in the tangential section. As shown in Fig. 4b, the pits along the vessel walls were obviously covered by oil in the bamboo samples after oil heat treatment at different temperatures. Moreover, the pits in vessels became to shrink as treatment temperature increased^34^. When the temperature reached 200 °C, the pits almost disappeared. The results implied that although heat treatment at high temperature up to 200 °C has little impact on the integral skeleton of bamboo, it was able to cause significant shrinkage of pits. The evaporation rate of moisture and moisture gradient in bamboo at high temperature would lead to the increase in the internal stresses of bamboo and the degree of pits deformation during oil heat treatment^34^. Moreover, the hemicellulose content significantly decreased as the temperature increased, which reduced from 22.92% for the untreated sample to 15.91% for bamboo sample after oil heat treatment at 200 °C (Table S3). Thus, the evaporation of moisture and the changes of chemical structure during oil heat treatment might contribute to the reduction of strength around pits and the increase of pit shrinkage. The closed or covered pits in the parenchyma cells and vessels in the treated samples would effectively inhibit external substances such as water, organism and fungus entering into the bamboo interior, thus protecting bamboo from mildew and deformation. Fluorescent staining method with CLSM was also employed to further investigate tung oil distribution in treated samples on the microscopic level. Here, Nile Red was used as the fluorescent agent to label tung oil and the samples were excited at 633 nm to avoid the auto-fluorescence of bamboo (Fig. S2). The results, shown in Fig. 5, supported that tung oil indeed distributed well in the cell walls of bamboo samples after oil heat treatment, rather than merely stored in the lumen and intercellular space.

Table S3 showed the results of chemical compositions of the bamboo samples before and after oil heat treatment. It can be observed that the hemicellulose content significantly decreased with increase of temperature, which reduced from 22.92% for the untreated sample to 15.91% for the bamboo sample after oil heat treatment at 200 °C. The relatively easy thermal degradation of hemicellulose might be attributed to the existence of many acetyl groups in the hemicellulose, which leads to the formation of acetic acid, thereby causing acid-catalyzed degradation of the polysaccharides^35^. For the cellulose, heat treatment in tung oil at high temperature below 180 °C did not result in reduction of cellulose content, which could be explained that cellulose has a semi-crystalline structure and there are limited accessible glucosidic bonds in cellulose^36^. In addition, the lignin content was slightly enhanced with increasing the heating temperature. As lignin is the most heat-resistant component in bamboo^37–39^, the slight increase in lignin content may be due to the condensation and cross-link reactions of lignin or production of compounds featuring aromatic ring products induced by heat treatment^7^.

The changes of chemical structure in the bamboo materials after tung oil heat treatment were also examined by Fourier transform infrared (FTIR) measurements. As shown in Fig. S3, compared with the untreated sample and the sample after heat treatment in air, the samples after oil heat treatment exhibited a new band at 3010 cm^−1^, attributed to C-H moieties at the carbon-carbon double bond in tung oil^40^. The results proved the presence of tung oil in the bamboo samples after oil heat treatment.

Besides that, the FTIR spectra of the samples after oil heat treatment featured characteristic bands of hemicellulose at around 1740 cm^−1^ and lignin at 1505 cm^−1^, which was similar to those of the untreated sample, suggesting that the original chemical structure of bamboo remained after oil heat treatment^21,41^. The band at 1730–1745 cm^−1^ was probably ascribed to the C=O stretching vibrations of acetyl and uronic ester groups in hemicellulose or ester linkage of carboxylic groups in lignin or ester groups in tung oil^42,43^. This band was reinforced after oil heat treatment at 100–140 °C. Combined with the chemical composition results (Table S3) that tung oil heat treatment at low temperature had little effect on the content of hemicellulose and lignin, the increased band was mainly due to the presence of tung oil in the oil treated bamboo. On the other hand, the intensity of the band at 1730–1745 cm^−1^ decreased with further increasing temperature, particularly at the high temperature of 180–200 °C. Take into account that the hemicellulose content was significantly decreased, the lignin content was slightly enhanced (Table S3), and oil uptake percentage was relatively decreased as the heating temperature increased from 180 °C to 200 °C (Table S1), the decreased band might be caused by both the degradation of acetyl groups in hemicellulose through deacetylation reactions and the decrease of ester groups in tung oil. The samples after oil heat treatment also showed stronger intensity of band at 1505 cm^−1^, assigned to C=C stretching of aromatic skeletal vibrations in lignin^44^, indicating an increase of lignin content after oil heat treatment. FTIR results suggested that the hemicellulose content decreased, while the content of lignin relative increased after oil heat treatment at high temperature.

To further analyze the cellulose structure and the crystallinity index (CrI) of bamboo samples after heat treatment in tung oil, X-ray diffraction (XRD) patterns were studied, and the results were shown in Fig. S4. Similar diffraction patterns of different samples were observed. However, after heat treatment in tung oil, the diffraction peak of 22.5° became sharper with the increase of heating temperature, suggesting that the bamboo samples became more crystalline. The CrI was calculated and the results were listed in Table S4. It was observed that the CrI enhanced from 24.5% for the untreated sample to 44.4% for the samples after oil heat treatment at 200 °C, confirming that heat treatment would result in more crystalline of cellulose. As mentioned above, during oil heat treatment, the partial hemicellulose in bamboo samples was degraded. Additionally, heat treatment also promoted the strands of molecules in amorphous cellulose to rearrange more orderly and closer together. Thus, the increasement of cellulose crystallinity was probably due to degradation of hemicelluloses and rearrangement of cellulose in the amorphous regions^38^.

### Thermal property

The thermal property was also evaluated by Thermogravimetric (TG) analysis. As shown in Fig. S5a, the TG curves of bamboo samples can be divided into three regions. The first region of 23–180 °C was associated with the evaporation of water in the bamboo and the release of a small amount of volatiles; the second region of 180–400 °C was mainly related to thermal degradation of hemicellulose, cellulose and partial tung oil; the depolymerization of hemicellulose mainly happened at 180–320 °C and the degradation of cellulose mainly occurred between 320 °C and 400 °C; in the third region of 400–800 °C, the weight loss was primarily due to the thermal decomposition of lignin in the bamboo. The results showed that the weight loss in region of 23–200 °C had a slight change between untreated sample and tung oil treated samples, ranging from 0.14% to 4.18%. The slight change of weight loss in the region of 23–200 °C might be due to the variation of moisture content of bamboo samples. In the region of 180–800 °C, the TG curves of tung oil treated samples were generally shifted to higher temperature (Fig. S5a), compared with the untreated sample, suggesting better thermal stability of bamboo samples after tung oil heat treatment.

Similarly, there were two peaks in DTG curves observed in Fig. S5b, the one at 290 °C being convoluted with the main peak at around 335 °C. The first peak at 290 °C was assigned to the thermal pyrolysis of hemicellulose. Compared to untreated sample, the intensity of the peak at 290 °C was progressively decreased as the temperature of tung oil heat treatment increased, which was related to the decrease of hemicellulose content. The second peak at around 335 °C was mainly attributed to the decomposition of cellulose. Careful observation of the DTG curves revealed that the main peak was shifted from 335 °C for untreated sample to 337 °C for 23 °C-Oil and 351 °C for 180 °C-Oil, respectively, supporting the better thermal stability of cellulose in the tung oil treated bamboo samples, mostly related with increasing crystallinity of cellulose^45^. Besides, the position of the main peak slightly decreased for 200 °C-Oil, perhaps reflecting degradation of the tung oil itself. Tung oil is thermally stable up to 200 °C and about 10% of weight loss would happen between 200–400 °C. The intensity of the main peak at around 335 °C for the samples after tung oil treatment was generally higher than the untreated sample, which might be influenced by both the increasing crystallinity of cellulose and the degradation of tung oil in the modified bamboo. Combined with the results of IR and chemical composition analysis, the improved thermal stability of bamboo samples after tung oil heat treatment was probably due to the partial degradation of amorphous carbohydrates, such as hemicellulose, and the increment of cellulose crystalline.

### Surface wettability

**Figure 6:**
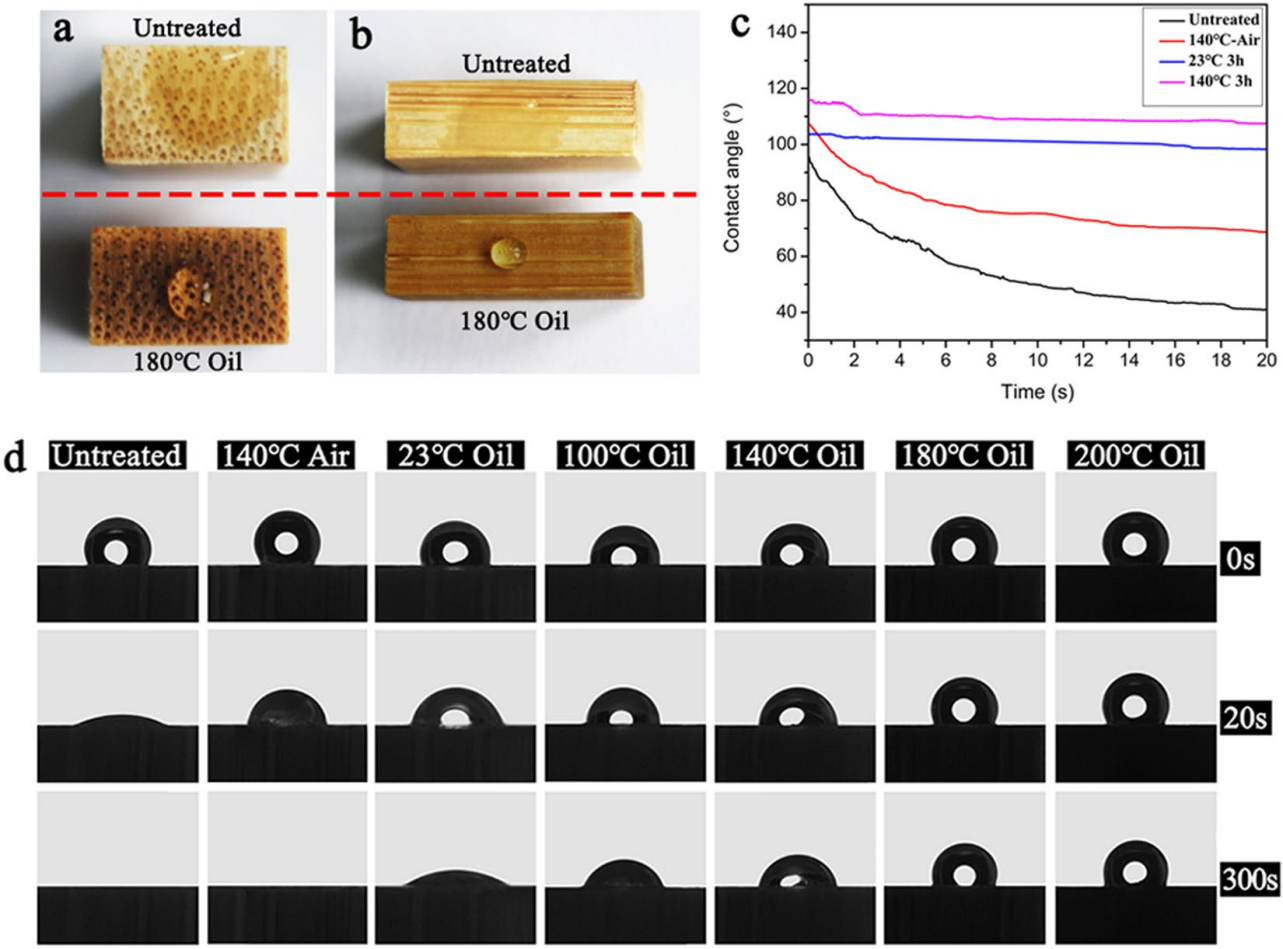
Wetting behavior of different bamboo samples. Images of a water droplet on the surface (**a**) in transverse section, (**b**) in radial section, (**c**) contact angle-time curve in radial section, (**d**) contact angles in transverse section.

Water contact angles were measured to analyze the effect of tung oil heat treatment on the surface hydrophobic properties of bamboo materials. As shown in Fig. 6, when distilled water dropped on the surface of the untreated sample, the water droplet was immediately absorbed into the sample, indicating poor surface hydrophobicity. For the sample after heat treatment in air and the sample after oil treatment at 23 °C, the initial contact angle in the transverse section was higher (>100°), but obviously decreased after 20 s, and further reduced to <5° after 300 s (Fig. 6d), which indicated that both heat treatment and tung oil modification were able to improve the short-lasting hydrophobic properties of bamboo. By contrast, the bamboo samples had a better and longer lasting hydrophobicity by synthetic effects of heat and tung oil. The hydrophobicity was enhanced with increasing the heating temperature, and the contact angles could keep >100° after 300 s for the samples after heat treatment in tung oil at 200 °C. The bamboo samples in radial section also had the similar hydrophobic properties to those in the radial section (Fig. S6).

**Figure 7:**
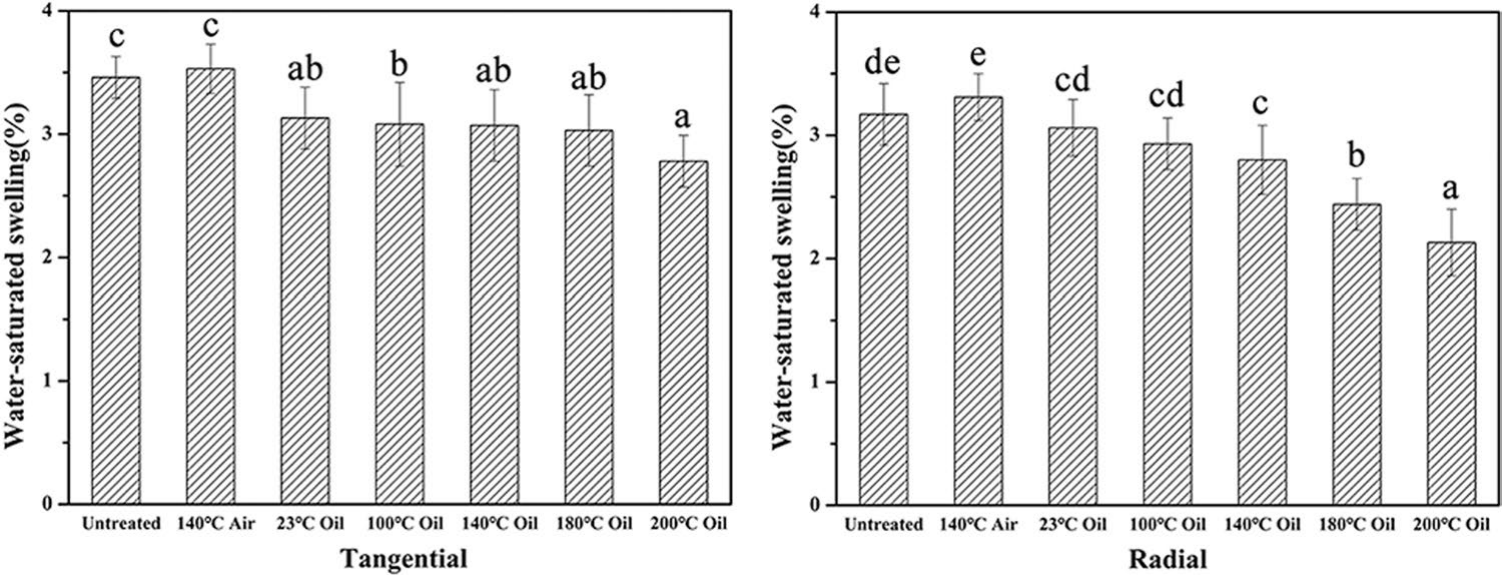
Anti-swelling efficiency of the bamboo samples at room temperature (**a**) tangential direction, (**b**) radial direction.

The long-term water resistance of bamboo materials was characterized by dimensional stability. The bamboo samples were firstly dried at 105 °C in the oven for 24 h, and then placed in distilled water with a temperature of 20 ± 2 °C for 30 days until they reached constant dimensions. The dimensions were measured at room temperature to obtain the water-statured swelling. As shown in Fig. 7b, the value of water-saturated swelling was gradually decreased with increasing heating temperature, which reduced from 3.17% for the untreated sample to 2.42% for the samples after oil heat treatment at 200 °C in the radial direction. As a comparison, the water-saturated swelling was slightly increased to 3.31% for the sample after heat treatment in air. In the tangential direction (Fig. 7a), the samples after oil heat treatment also showed a better dimensional stability than the air heat treated and the untreated samples. Combined with the above results, the samples after oil heat treatment were demonstrated to have excellent hydrophobic properties and dimensional stability.

As reported before, tung oil has an excellent water repellence^46^. Combined with the SEM results, bamboo contains numerous pits on the cell walls, connecting adjacent tissue (Fig. 1). After oil heat treatment, the cell walls of bamboo samples were covered with an oily film and most of the pits were blocked with tung oil (Fig. 4), so that water transport pathways were curbed. Furthermore, oil heat treatment at high temperature such as 200 °C caused the shrinkage and blocking of pits, which are the important factors contributing to the improvement of hydrophobic property and dimensional stability. In addition, tung oil heat treatment effectively reduces the water affinity groups content, enhances the inaccessibility of hydroxyl groups to water molecules for increment of cellulose crystallinity, and promotes further crosslinking by the polycondensation reactions in lignin, which are also important factors that attribute to the improvement in water resistance of the modified bamboo^47^.

### Fungi resistance

**Figure 8:**
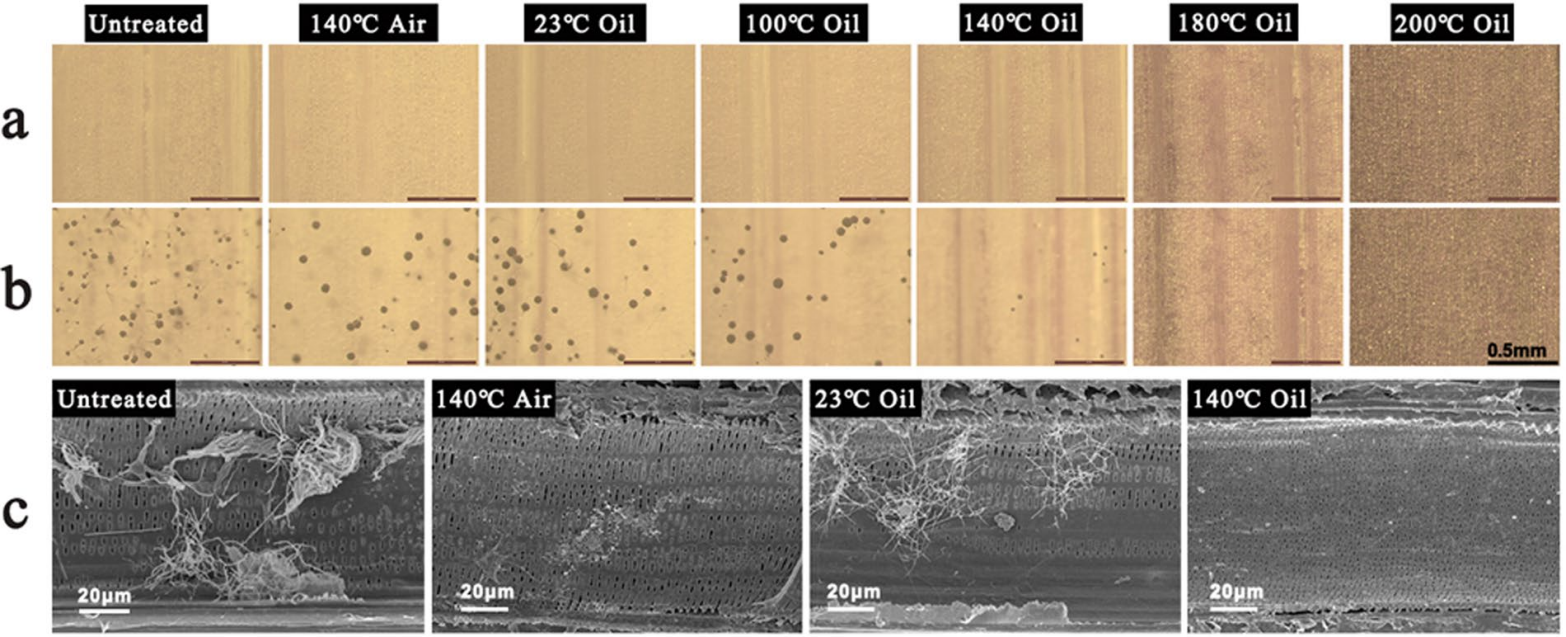
Fungi resistance of bamboo (**a**) microscope images of bamboo samples, (**b**) microscope images of bamboo samples after exposed to Aspergillus niger for 3 days, (**c**) SEM images of bamboo after exposed to Aspergillus niger for 8 weeks.

Although bamboo was used as furniture and building decoration materials for thousands of years, it was largely overtaken by man-made materials in the outdoor materials, due to the lack of fungi resistance. Improvement in fungi resistance such as Aspergillus niger is one of the advantages of heat treated bamboo with tung oil. Through microscope observations (Figs 8 and S7), the surface of the untreated sample was completely covered by the fungi, while the samples after oil heat treatment were less infected after exposure to Aspergillus niger for 3 days. When the samples were treated in tung oil at high temperature above 180 °C, few fungi were observed on the bamboo surface, indicating that tung oil heat treatment could effectively improve the fungi resistance of bamboo. To further investigate the effect of tung oil heat treatment on the bamboo against Aspergillus niger, the samples were exposed to Aspergillus niger for 8 weeks, and then SEM was used to observe the details of Aspergillus niger growth inside the bamboo. From the SEM images (Fig. 8c), it can be seen that the vessels of the untreated sample were invaded by massive Aspergillus niger, while the fungi quantity in the sample after heat treatment in air and the sample after oil treatment at 23 °C was less than that in the untreated sample. For the sample after oil heat treatment, few Aspergillus nigers were found on the walls of vessels, further demonstrating that both tung oil and heat treatment help the bamboo samples improve the property of fungi resistance. As hemicellulose is considered as an important nutritive source of fungi^16^. After heat treatment in tung oil, the decrease of hemicellulose content and moisture content in bamboo samples can inhibit the growth of fungi^48^. Besides, the formed oily films in the surface and inside of bamboo samples also effectively prevent water and fungi from penetrating into the inside of bamboo, thus leading to the improvement of the property of fungi resistance.

### Mechanical property

The mechanical properties of bamboo materials were tested by three point bending method and the results were shown in Fig. S8. It was observed (Fig. S8d) that heat treatment of bamboo in tung oil at <140 °C did not affect the breaking toughness significantly, until the temperature reached over 180 °C. It is known that the connection of glucomannan in hemicelluloses to the surface of the cellulose is mainly based on hydrogen bonds, while, to some extent, lignin is covalently bound to hemicelluloses^49,50^. The chemical composition results (Table S3) showed that partial hemicellulose were degraded by tung oil heat treatment, resulting in weakening the flexible connections among the different composition in the cell walls, which might be the main reason for the decrease in breaking toughness. Heat treatment in tung oil below 200 °C did not reduce the bending strength of bamboo. By contrary, both the modulus of elasticity (MOE) and modulus of rupture (MOR) were improved when the temperature was below 140 °C. The improvement was probably produced by higher density and cellulose crystallinity^51,52^. Even increasing the temperature up to 200 °C, MOE and MOR kept at 10.1 GPa and 126.9 MPa, respectively, which were not lower than those of the untreated sample (9.2 GPa, 125.3 MPa). As a higher MOE indicates that the bamboo material is stiffer and has better dimensional stability, while MOR represents the amount of stress that bamboo can withstand. The results suggested that bamboo materials after heat treatment in tung oil below 200 °C maintained satisfactory mechanical performance. Therefore, the bamboo materials after tung oil heat treatment could be widely applied in the indoor and outdoor such as floorings, furniture and fencing.

## Conclusions

In summary, we have shown that heat treatment in tung oil is an effective approach to modify bamboo to produce more durable bamboo materials. When bamboo materials were thermally treated in tung oil, tung oil primarily permeated through the longitudinal direction by vascular bundles, and then diffused into the interior by pits. Tung oil can be distributed evenly in the cell walls of bamboo samples after oil heat treatment. The chemical composition, cellulose crystalline structure and performance of moso bamboo were greatly influenced by oil heat treatment. Heat treatment in oil would reduce hydrophilic group content as a result of thermal degradation of amorphous carbohydrates such as hemicellulose, and increase cellulose crystallinity by rearrangements in the crystalline region of cellulose. Moreover, oily films were formed inside and over the bamboo surface to protect bamboo materials from water, fungi and deformation. As a result, heat treatment in tung oil not only enhanced the hydrophobic property and dimensional stability of bamboo, but also brought about a considerable improvement in fungi resistance. It is worth noting that although heat treatment of bamboo in tung oil at high temperature over 180 °C induced an obvious reduction in breaking toughness, bamboo material after heat treatment in tung oil below 200 °C still maintains good mechanical performance with no obvious drop in MOE and MOR, compared to the untreated bamboo. Therefore, tung oil heat treatment could be used as an economical and eco-friendly method to modify bamboo materials, which is highly promising in the bamboo industry.

## Supplementary information

Supplementary Information
