# Supplementary material for: Synergistic effects of tung oil and heat treatment on physicochemical properties of bamboo materials
Source: Sci Rep. 2019 Sep 6;9:12824. doi: 10.1038/s41598-019-49240-8 (PMC6731322; doi:10.1038/s41598-019-49240-8)
Supplement: Supplementary file 1 — Supplementary Information [file 41598_2019_49240_MOESM1_ESM.doc]

**Electronic Supplementary Information**

**Synergistic effects of tung oil and heat treatment on physicochemical properties of bamboo materials**

Tong Tang^1,2^, Bo Zhang^2^, Xianmiao Liu^1^, Wenbo Wang^3^, Xiufang Chen^2,*^, Benhua Fei^1,*^

^1^ Key Laboratory of Bamboo and Rattan Science and Technology of the State Forestry Administration, Department of Bio-materials, International Centre for Bamboo and Rattan, Futong Dong Dajie, Chaoyang District, Beijing 100102, China

^2^ Qingdao Institute of Bioenergy and Bioprocess Technology, Chinese Academy of Sciences, Songling road, Qingdao 266101, Shandong, China

^3^ Research Institute of Forestry, Chinese Academy of Forestry, Xiangshan Road, Haidian District, Beijing 100091, China

^*^ Correspondence and requests for materials should be addressed to X.C. (Email: chenxf@qibebt.ac.cn) or B.F. (feibenhua@icbr.ac.cn)

Tel.: +86-010-84789788

Fax: +86-010-84789717

**Materials and experimental methods**

**Materials.** A 5 years old Moso bamboo (Phyllostachys heterocycla) was obtained from Xuancheng, Anhui, China. Moso bamboo from 1.5 m (height from base) to 3.5 m height was used in this study. Defect-free bamboo materials were dried at room temperature and cut from the center region to three sizes: 100 × 5 × 5 mm^3^, 20 × 20 × 5 mm^3^, and 20 × 5 × 5 mm^3^ (longitudinal × tangential × radial). The process of samples preparation was shown in figure S1. Then samples were kept in a climate- controlled room until the moisture content of approximately 12% before use. Tung oil was purchased from Emperor's craftsman, Shanghai, China. The tung oil mainly contains about 82% ofα-eleostearic acid, 8.5% of oleic acid and 9.5% of linoleic acid. The density of tung oil is about 0.937 g/mL and the viscosity is 764.6 MPa. The boiling point of tung oil is about 235 ℃ (at 1.6 KPa). Sudan black (Aladdin Co., Ltd., Shanghai, China) and Nile Red (Sigma-Aldrich Co., Ltd., MO, USA) used to stain oil. All chemicals were used as received without further purification.

**Preparation of tung oil heat treatment bamboo samples.** Moso bamboo samples were immersed in tung oil at 100 ℃, 140 ℃, 180 ℃, 200 ℃ for 3 h, respectively. During heat treatment of samples, the operating temperature was maintained constant within ± 2 ℃. After oil heat treatment, the samples were wiped and then conditioned in a climate-controlled room (at 65 ± 5% relative humidity and temperature of 20 ± 2 ℃) to reach equilibrium moisture content. As a comparison, the samples were immersed in tung oil at 23 ℃ for 3h or heat treated at 140 ℃ in the air for 3h.

**Characterization.** The morphology of bamboo was characterized by scanning electron microscopy (SEM) (Model XL30, Fei, USA). Fourier transform infrared (FTIR) spectra were recorded on a spectrometer (Nicolet iN10, Thermo Scientific, USA) over the wavenumber range of 500-4000 cm^-1^ at a resolution of 4 cm^-1^, using the KBr pellet method. The X-ray diffractograms (XRD) were performed by X-ray diffractometer (D8 Advance, Bruker, Germany). The thermogravimetric (TG) analysis was performed under nitrogen atmosphere using thermal analyzer (STA449F5 Jupiter, NETZSCH, Germany). Elemental components (C, H and O) of bamboo were determined on an elemental analyzer (Vario EL, Elementar, Germany). The chemical components of bamboo were analyzed according to the methods of US National Renewable Energy Laboratory (NREL)[^1^](#_ENREF_24). The acid soluble lignin fraction in the solution was determined by UV−vis spectrometer (752N, jingke, China), and sugar (glucose, xylose, and arabinose) fractions in hydrolysate were analyzed by High Performance Liquid Chromatography (HPLC) (1200series, Agilent, USA) and calibrated using standard sugars.

**Oil permeation behavior.** A heat resistant dyed oil was prepared by dissolving 1g of the fat soluble and the heat resistant stain Sudan Black in 1L of the tung oil[^2^](#_ENREF_24). Tung oil mixed with the Sudan black was heated and stirred over a hot plate at 60 ℃ for 4 h until the stain was dissolved completely. Then, the oil temperature was heated up to 140 ℃, and bamboo samples were immersed in oil for different times to investigate the permeation behavior of tung oil.

**Oil distribution at the micro-scale.** Nile red was dissolved in tung oil with a concentration of 0.0192 mg/mL[^3^](#_ENREF_24). Bamboo sample was heat treated in the tung oil solution at 140℃ for 3h. Then the 10 μm thick of bamboo sample were cut by a sliding microtome and rapidly covered with a coverslip on a clean glass slide. The confocal laser scanning microscope (GLSM) (FV1000, Olympus, Japan) was used to investigate tung oil distribution in bamboo. The sample was excited at 633 nm to avoid the auto-fluorescence of bamboo.

**Weight percentage gain (WPG).** Moso bamboo samples (percentage of moisture were 12%) were weighed before modification (W_1_). The modified samples were weighted again (W_2_). The weight percentage gain can be calculated.

**Density.** The density of bamboo was measured using the water displacement method. Samples were weighed, dried in an oven at 103 ± 2 °C overnight and the dry mass were measured. The density can be calculated. The measurements were performed for more than ten times.

**Contact angles.** The contact angles of the bamboo samples were measured by a contact angle measurement system (OCA 20, Data Physics, Germany) with 4 μL water droplets. The contact angles were measured 0 s, 10 s, 20 s and 300 s after the contact of the drop with the specimens, and the average angles were calculated to determine the wettability. The measurements were performed for more than five times.

**Dimensional stability.** From each group, ten samples with a size of 20 × 20 × 5 mm^3^ (longitudinal × tangential × radial) were used to measure swelling efficiency of bamboo samples. The bamboo samples were firstly dried at 105 ℃ in the oven for 24 h and placed in distilled water with a temperature of 20 ± 2 ℃ for 30 days until they reached constant dimensions. Then the dimensions were measured to obtain water-statured swelling under the room temperature condition. The dimensions were measured in two directions (tangential and radial). The measurements were repeated ten times.

**Fungi resistance.** The bamboo samples were placed in Aspergillus niger at 37 °C and 80% relative humidity for 8 weeks. Visual aspect of the Aspergillus niger colony on the surface of bamboo samples was monitored by stereoscope instrument (DFC425C, Leica, Germany) every day, during the first week of incubation. After the samples were exposed to Aspergillus niger for 8 weeks, they were taken out from the culture plates, and the mycelium present on the surface was removed. Then, the bamboo samples were investigated by SEM. The measurements were performed for more than five times.

**Bending strength.** The moso bamboo with a size of 100 × 5 × 5 mm^3^ (longitudinal × tangential × radial) was analyzed using a three-point bending test by an Instron Microtester (5848, Instron, USA) to measure the stress and strain[^4^](#_ENREF_24). The measurements were repeated ten times. Three-point bending set-up was used with a span of 80 mm and a crosshead speed of 4 mm per min. The moisture percentage of the bamboo samples were about 12%.

**References**

1. Sluiter, A., *et al.* Determination of structural carbohydrates and lignin in biomass. Laboratory Analytical Procedure (LAP) NREL/TP-510-42618; National Renewable Energy Laboratory: Golden, CO (2011).
2. Pedreschi, F., Cocio, C., Moyano, P. & Troncoso, E. Oil distribution in potato slices during frying. *J. Food Eng.* **87,** 200 (2008).
3. Jia, B., Fan, D., Li, J., Duan, Z. & Fan, L. Effect of guar gum with sorbitol coating on the properties and oil absorption of french fries. *Int. J. Mol. Sci.* **18,** 2700 (2017).
4. Wang, J.P., *et al.* Improving wood properties for wood utilization through multi-omics integration in lignin biosynthesis. *Nat. Commun.* **9,** 1579 (2018).

**Figure S1** The process of sample preparation.


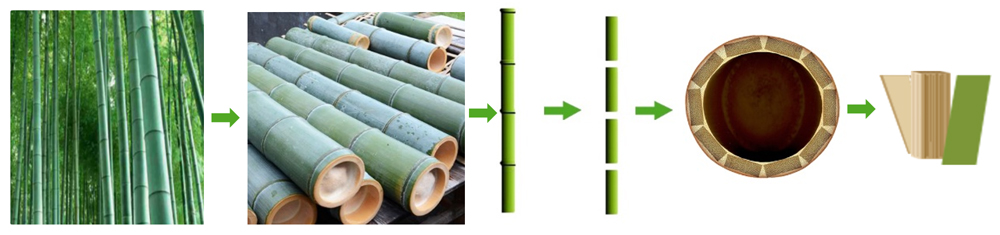


**Figure S2** Fluorescence spectra of bamboo (a) untreated bamboo, (b) oil heat treated bamboo.

**
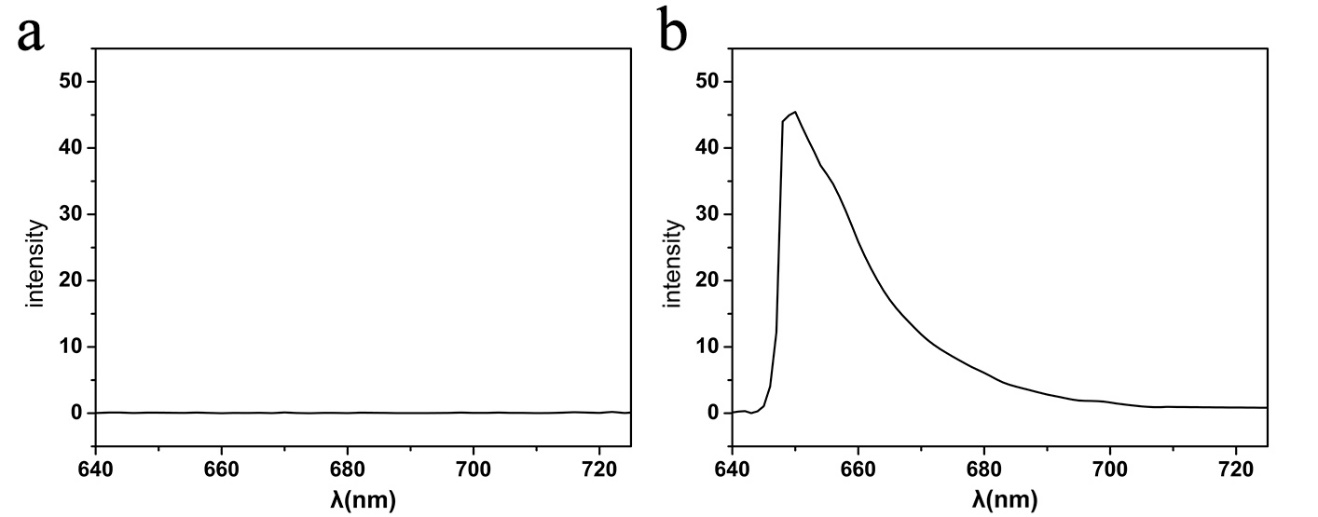
**

**Figure S3** FTIR spectra of different bamboo samples.


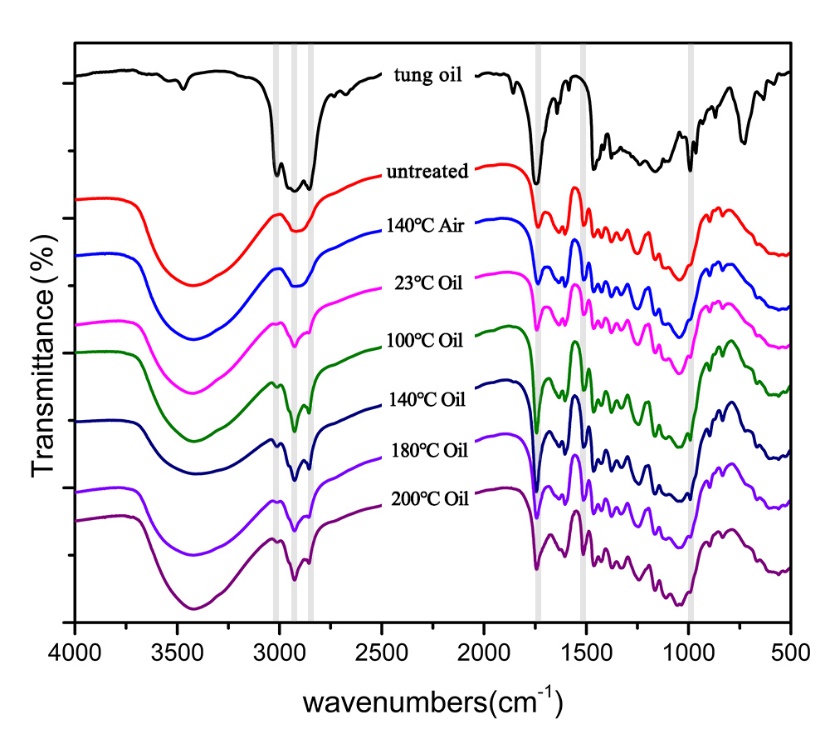


**Figure S4** XRD patterns of different bamboo samples.


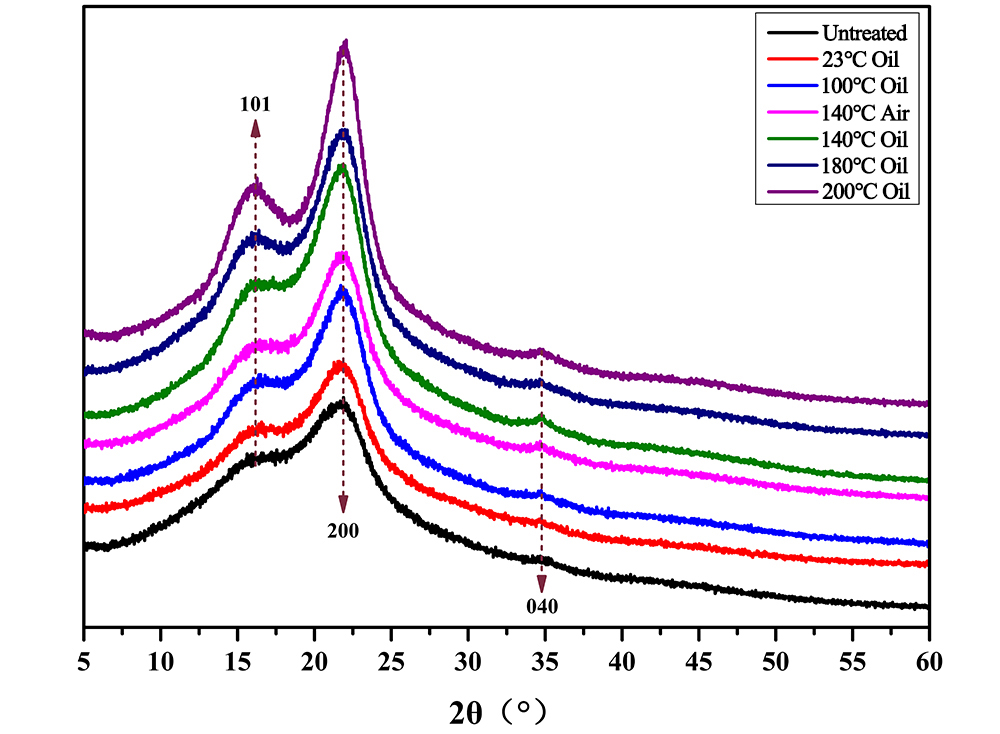


**Figure S5** TG and DTG curves of different bamboo samples.


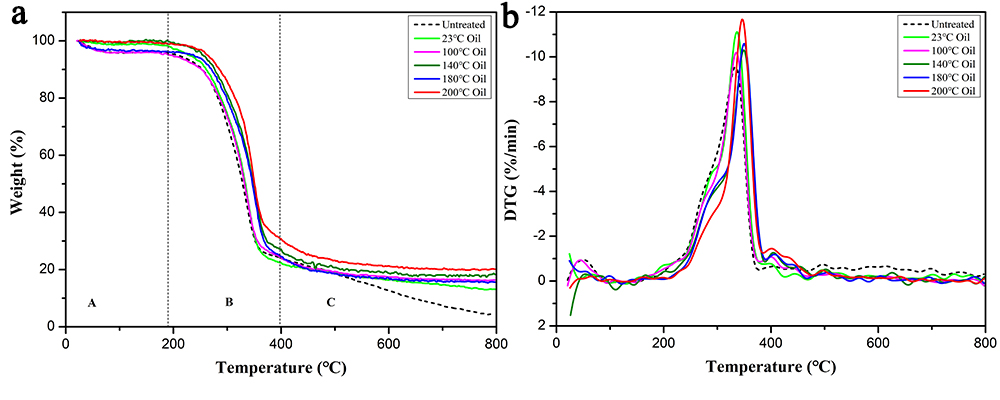


**Figure S6** Contact angles of different bamboo samples.


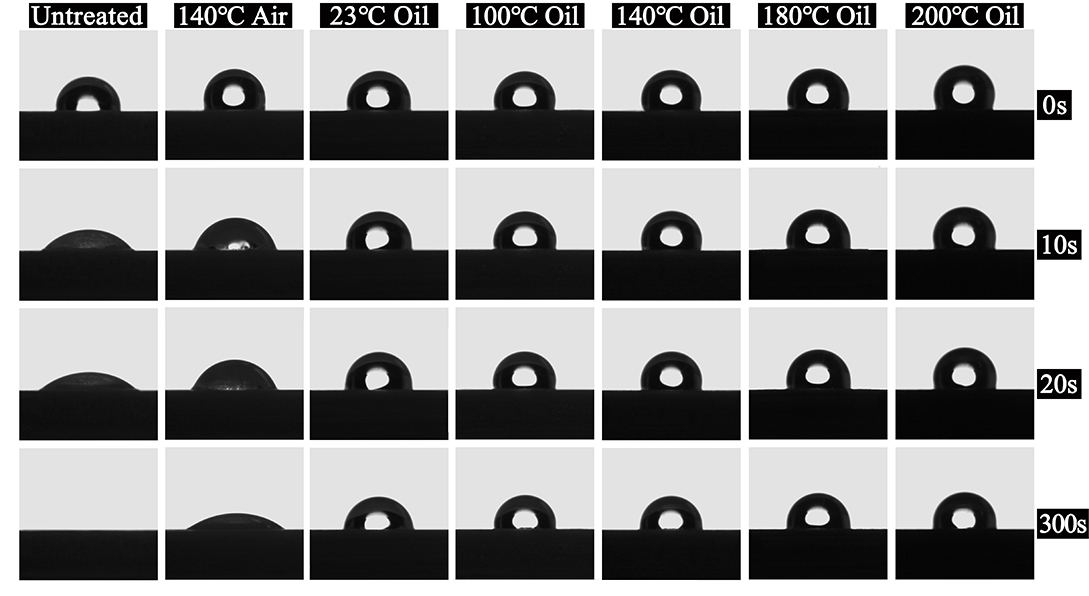


**Figure S7** Fungi resistance of bamboo samples after exposure to Aspergillus niger for 28 days.


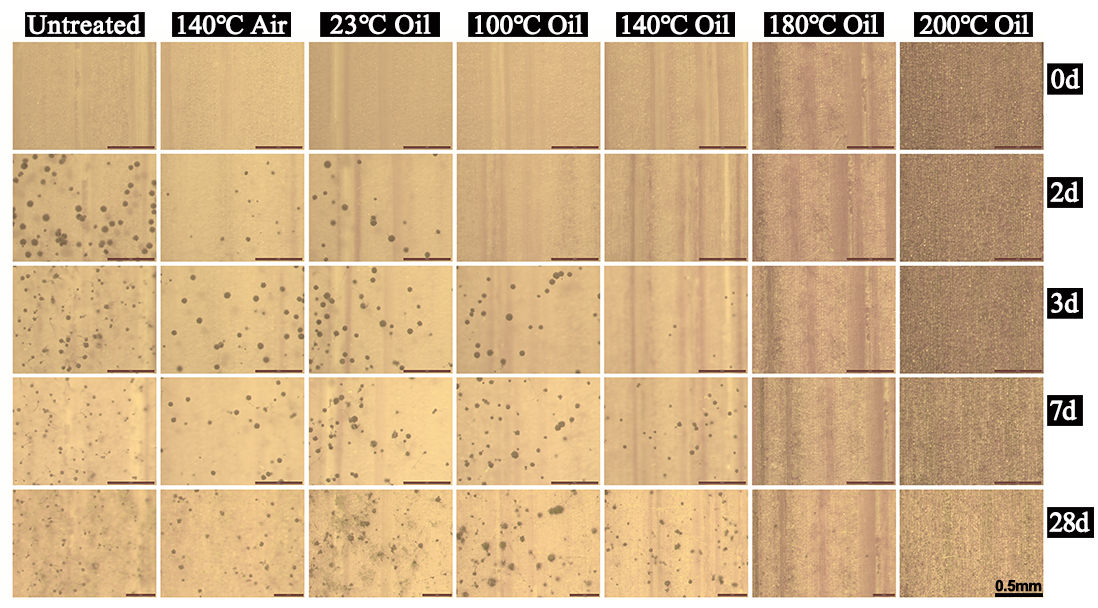


**Figure S8** Physical and mechanical properties of different bamboo samples on radial section. (a) MOE, (b) MOR, (c) density, (d) bending stress-strain curves.


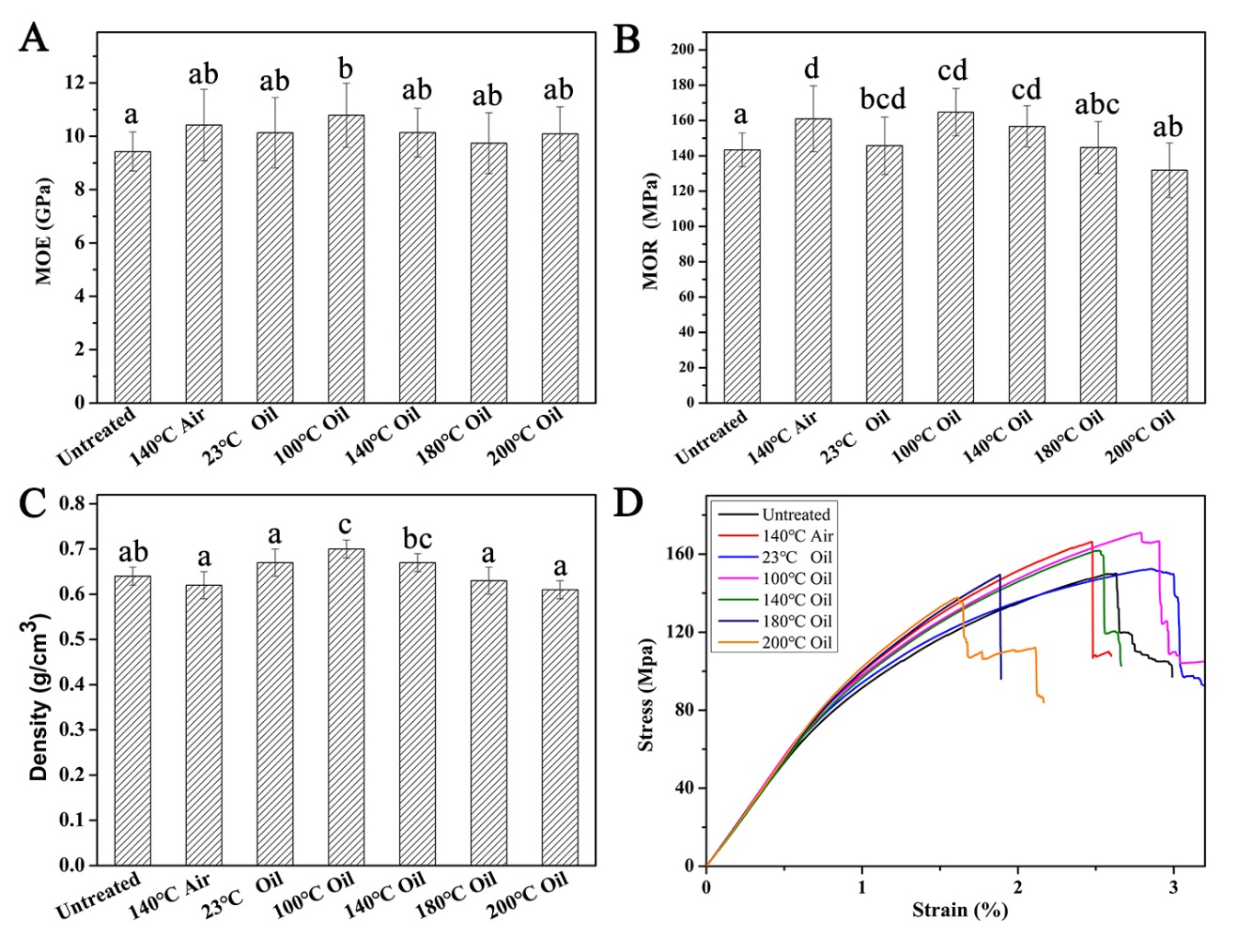


**Table S1** WPG of the bamboo samples after heat treatment in air and tung oil.

| Treatment (℃) | WPG (%) | |
| --- | --- | --- |
|  | Air | Tung oil |
| 23 | - | 3.81^d^ (0.26) |
| 100 | -9.54 ^d^ (0.34) | 3.10 ^d^ (0.47) |
| 140 | -10.03 ^c^ (0.14) | 2.00 ^c^ (0.45) |
| 180 | -10.96 ^b^ (0.23) | -4.69 ^b^ (0.88) |
| 200 | -14.73 ^a^ (0.17) | -9.25 ^a^ (1.51) |

Mean values followed by the same superscript letters in the same column are not significantly different at p＜0.05. Values in parentheses are standard deviations.

**Table S2** Element content of different bamboo samples..

| Treatment (℃) | Transfer Medium | C (wt %) | O (wt %) | H (wt %) | O/C (at/at) |
| --- | --- | --- | --- | --- | --- |
| untreated | - | 42.62 | 43.23 | 9.44 | 0.76 |
| 140 | Air | 42.48 | 44.09 | 10.35 | 0.78 |
| 23 | Tung Oil | 43.61 | 43.62 | 9.50 | 0.75 |
| 100 | Tung Oil | 45.44 | 42.62 | 13.05 | 0.70 |
| 140 | Tung Oil | 45.80 | 42.22 | 10.37 | 0.69 |
| 180 | Tung Oil | 46.85 | 41.27 | 11.72 | 0.66 |
| 200 | Tung Oil | 48.28 | 38.97 | 10.88 | 0.61 |

**Table S3 Chemical composition of different bamboo samples.**

| Heat Treatment (℃) | Cellulose (%) | Hemicellulose (%) | Lignin (%) | Extractives (%) |
| --- | --- | --- | --- | --- |
| untreated | 35.20^ab^ | 22.92^cd^ | 28.03^b^ | 8.48^b^ |
|  | (0.73) | (0.07) | (0.52) | (0.13) |
| 23 | 35.64^bc^ | 22.98^d^ | 26.73^b^ | 9.16^c^ |
|  | (0.50) | (0.63) | (0.26) | (0.32) |
| 100 | 31.88^a^ | 22.39^d^ | 28.49^d^ | 11.87^f^ |
|  | (0.22) | (1.09) | (0.01) | (0.26) |
| 140 | 33.90^b^ | 20.80^bc^ | 28.71^d^ | 11.13^e^ |
|  | (1.44) | (1.06) | (0.24) | (0.16) |
| 180 | 35.19^b^ | 19.87^b^ | 28.94^c^ | 10.58^d^ |
|  | (0.64) | (1.17) | (0.14) | (0.26) |
| 200 | 36.99^c^ | 15.91^a^ | 29.22^c^ | 13.33^g^ |
|  | (0.15) | (0.13) | (0.18) | (0.55) |

Mean values followed by the same superscript letters in the same column are not significantly different at p＜0.05. Values in parentheses are standard deviations.

**Table S4** Crystallization index of different bamboo samples.

| Heat Treatment (℃) | Transfer Medium | Crystallinity index (%) |
| --- | --- | --- |
| Untreated | - | 24.5 ^a^ |
| 140 | Air | 33.1 ^ab^ |
| 23 | Tung Oil | 27.6 ^ab^ |
| 100 | Tung Oil | 33.3 ^ab^ |
| 140 | Tung Oil | 34.0 ^ab^ |
| 180 | Tung Oil | 35.5 ^bc^ |
| 200 | Tung Oil | 44.4 ^c^ |

Mean values followed by the same superscript letters in the same column are not significantly different at p＜0.05. Values in parentheses are standard deviations.
